# Passband switchable microwave photonic multiband filter

**DOI:** 10.1038/srep15882

**Published:** 2015-11-02

**Authors:** Jia Ge, Mable P. Fok

**Affiliations:** 1 Lightwave and Microwave Photonic Laboratory, College of Engineering, The University of Georgia, USA.

## Abstract

A reconfigurable microwave photonic (MWP) multiband filter with selectable and switchable passbands is proposed and experimentally demonstrated, with a maximum of 12 simultaneous passbands evenly distributed from 0 to 10 GHz. The scheme is based on the generation of tunable optical comb lines using a two-stage Lyot loop filter, such that various filter tap spacings and spectral combinations are obtained for the configuration of the MWP filter. Through polarization state adjustment inside the Lyot loop filter, an optical frequency comb with 12 different comb spacings is achieved, which corresponds to a MWP filter with 12 selectable passbands. Center frequencies of the filter passbands are switchable, while the number of simultaneous passbands is tunable from 1 to 12. Furthermore, the MWP multiband filter can either work as an all-block, single-band or multiband filter with various passband combinations, which provide exceptional operation flexibility. All the passbands have over 30 dB sidelobe suppression and 3-dB bandwidth of 200 MHz, providing good filter selectivity.

Multiband communications have been extremely important for modern wireless communications and satellite communications in the commercial, defense, and civilian federal marketplace^1^,^2^,^3^,^4^. The driving forces behind this are the increasing demand for multifunctional devices, the use of cognitive wireless technology to solve the frequency resource shortage problem, as well as the capabilities and operational flexibility necessary to meet ever-changing requirements. Thus, to pre-select the desired band, and prevent interference in multiband communications, tunable multiband RF filters are critically needed. Unfortunately, this kind of RF bandpass filter is difficult to achieve using conventional RF electronic techniques because of the lack of reconfigurability; in addition, it is also difficult to simultaneously satisfy all the design parameters for all passbands^5^,^6^,^7^,^8^,^9^,^10^. An example of a RF multiband filter with six passbands is demonstrated based on the use of several cascaded resonators. While it does offer an on-chip solution, the scheme is inflexible with a limited number of passbands of six, and it is hard to obtain passbands with consistent filter profiles^11^. Microwave photonic (MWP) RF filters have received increasing attention in recent years due to the significant improvements over conventional RF filter, such as low-loss, wide bandwidth, flexibility, tunability and reconfigurability^12^,^13^,^14^,^15^,^16^,^17^,^18^,^19^,^20^,^21^,^22^. Different approaches for designing MWP bandpass filters have been reported frequently, including multi-tap delay line schemes^23^,^24^,^25^,^26^,^27^ and optical frequency comb schemes^28^,^29^,^30^,^31^,^32^,^33^,^34^,^35^,^36^,^37^,^38^,^39^. However, most existing approaches either lack of the ability to support multiband operations, or the resultant passbands are periodic over a very wide frequency range, limiting its ability to isolate unwanted frequencies within a certain frequency range. In order to achieve multiple passbands with a MWP filter, the corresponding optical frequency comb must either simultaneously consist of multiple combs with different comb spacings^38^ or it has to be sampled spectrally^39^. Unfortunately, due to the scalability, uniformity and selectivity of the above schemes, it is hard to achieve a MWP multiband filter with a large passband number, high selectivity, reconfigurable filter characteristics, and good passband uniformity. For example, a MWP multiband bandpass filter based on a high-birefringence loop mirror filter is proposed^38^. Three passbands with non-uniform bandwidths are achieved using three pieces of birefringence fibers, while a MWP multiband bandpass filter with sidelobe suppression of 10 dB is achieved with wavelength sampling technique^39^.

In this paper, a passband switchable MWP multiband filter based on the use of a two-stage Lyot loop filter is proposed and experimentally demonstrated. The two-stage Lyot loop filter is used to spectrally slice a broadband light source, such that optical frequency combs with 12 selectable comb spacing combinations are generated using just two pieces of polarization maintaining fiber. One or more of the comb spacings can be selected at the same time and the selected combs are spectrally interleaved in the resultant optical comb profile. With the use of the tunable two-stage Lyot loop filter for generating multi-taps of the MWP filter, a MWP multiband filter with switchable passband numbers and center frequencies is achieved. As a result, the MWP multiband filter has three operating states, i.e. single-band state, multiband state, and all-block state. In the single-band state, the MWP filter has only one passband and can be tuned to 12 different frequencies. While in the multiband state, up to 12 passbands within the frequency band of interest are observed at the same time. Both the passband numbers and frequencies are flexibly tuned by adjusting the polarization state in the two-stage Lyot loop filter. To the best of our knowledge, this is the first demonstration of a MWP multiband filter with such a large passband count, reconfigurable capability, uniform filter profile, and high selectivity. Moreover, sidelobe suppression of the passbands in the single-band state is up to 40 dB, and each of the passband shows a >30 dB sidelobe suppression in the multiband state. All the passbands have sharp and clean filter profiles as well as uniform 3-dB bandwidths of 200 MHz.

## Two-Stage Lyot Loop Filter with 12 Selectable Comb Spacings

**Figure 1:**
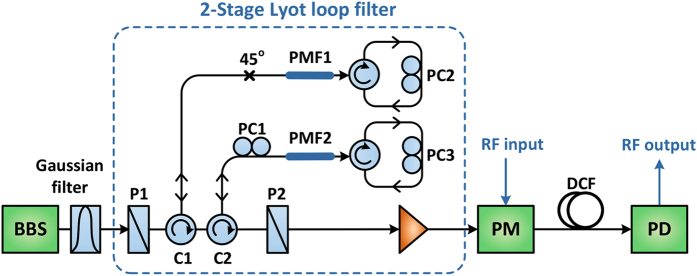
Experimental setup of the passband switchable MWP multiband filter. BBS: broadband source; P1–P2: polarizers; C1–C2: circulators; PC1–PC3: polarization controllers; PMF1–PMF2: polarization maintaining fibers; PM: phase modulator; DCF: dispersion compensating fiber; PD: photodetector.

Figure 1 shows the experimental setup of the proposed MWP multiband filter, where the two-stage Lyot loop filter is shown in the dashed box. A broadband amplified spontaneous emission (ASE) source is used as the light source and reshaped by an optical filter with a Gaussian profile. The reshaped broadband source is then launched into a two-stage Lyot loop filter via polarizer P1 for spectral slicing. Polarizer P2 is aligned with P1 like a classic Lyot filter^40^. At the output of P2, an optical comb source is generated and works as a multi-wavelength optical carrier for electrical-to-optical conversion, which is then modulated by the RF input signal through a phase modulator. Each of the comb lines generated by spectral slicing works as a single tap for the MWP filter, while the Gaussian optical filter is used to apodize the amplitude of the taps such that the MWP filter sidelobes can be greatly suppressed, resulting in a clean bandpass profile with high sidelobe suppression. The modulated signal is then launched into a piece of dispersion compensating fiber (DCF), which provides constant time delay between each filter taps. That is to say, each of the taps are weighted by the Gaussian filter and delayed by the DCF. The weighted and delayed signal is then fed into a photodetector and converted back to a RF signal. In the experiment, a sweeping RF signal from a network analyzer is used as the RF input for measuring the frequency response of the proposed MWP multiband filter.

**Figure 2:**
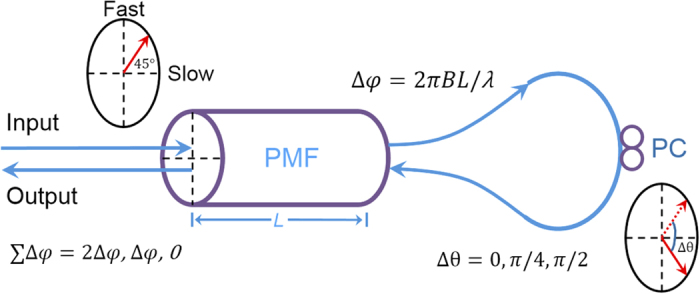
Operation principle of the comb spacing tunable Lyot loop filter using bidirectional propagation of light in a PMF. Input light propagates through a polarization maintaining fiber (PMF) twice bi-directionally via the circulator-PC loop (on the right) with a polarization rotation angle of 0, π/4 or π/2, resulting in a total phase difference of 2Δφ, Δφ or 0 at the output.

The two-stage Lyot loop filter used in the MWP filter is shown in the dashed box in Fig. 1. Each stage consists of a piece of polarization maintaining fiber (PMF), two polarization controllers (PC) and two optical circulators. The operating principle of each stage is illustrated in Fig. 2. Unlike the standard Lyot filter, light propagates through the PMF twice bi-directionally through a circulator-PC loop in our Lyot loop filter, which significantly increases the number of comb spacing combinations. In the Lyot loopes filter, a phase difference of 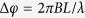 is obtained between the fast and slow axis when the light passes through the PMF at 45° (*π/*4) with respect to the fast axis, where *B* and *L* are the birefringence and length of the PMF, respectively, and *λ* is the wavelength of the light. By allowing the light to propagate twice in the PMF and adjusting the PC inside the loop to let the light have different polarization rotation angles (Δθ) of 0, *π/*4 or *π/*2 at the circulator-PC loop, a total phase difference (∑Δφ) of 2Δφ, Δφ and 0 can be obtained at the output, respectively. With different polarization rotation angles Δ*θ*, each stage works as one piece of PMF with an adjustable equivalent length (*L*_*e*_) of 2L, L and 0, correspondingly. With a two-stage Lyot loop filter, 12 different equivalent lengths (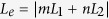, with m, n = 0, 1, or 2, shown in Table 1) are obtained, where *L*_*1*_ and *L*_*2*_ are the physical lengths of PMF1 and PMF2, respectively. As a result, a comb filter with 12 selectable comb spacings is achieved, in which the comb spacing (Δ*ω*, in angular frequency) is determined by the equivalent length of PMF, as shown in equation (1).


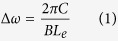


The comb spacing of the Lyot loop filter determines the carrier wavelength for each of the taps in the MWP filter, which in turn determines the temporal delay between taps after propagating in the DCF. By apodizing the tap amplitude with a Gaussian optical filter, a bandpass response in the RF domain with good sidelobe suppression and a clean passband profile can be achieved. The passband frequency (*Ω*_*0*_) of the MWP filter is governed by equation (2), where *β*_*2*_ and *L*_*D*_ are the group velocity dispersion and length of the DCF, which are fixed during the experiment. That is to say, the passband frequency is mainly governed by the optical comb spacing, which is tunable by adjusting the PC in the Lyot loop filter to change the Δ*θ* in Fig. 2. The 3-dB bandwidth of the passbands is determined by equation (3), where *δω* represents the overall bandwidth of the Gaussian optical comb^28^,^30^. As shown, the bandwidth of the MWP filter (*δΩ*_*3dB*_) is inversely proportional to the overall bandwidth of the optical comb and the length of the DCF. With the use of a longer piece of DCF, much narrower and further separated passbands can be obtained to meet specific requirements for different applications.


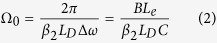



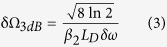


**Figure 3:**
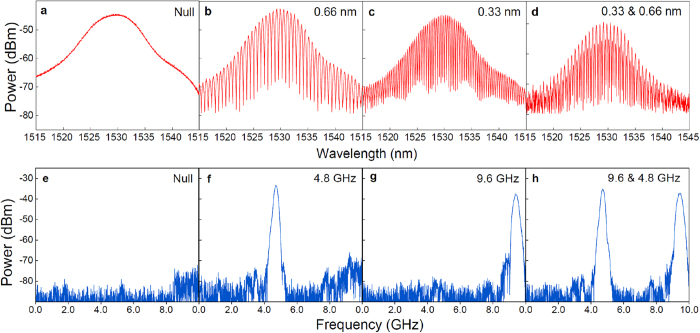
Measured optical comb spectra and frequency response of the one-stage Lyot loop filter based MWP multiband filter. (**a–d**) Gaussian optical frequency comb with different comb spacings: (**a**) no comb; (**b**) 0.66 nm; (**c**) 0.33 nm; (**d**) interleaved 0.33 nm & 0.66 nm. (**e–h**) Corresponding frequency response of the MWP multiband filter in 3 different operating states: (**e**) all-block state with zero passband; (**f**) single-band state with a passband at 4.8 GHz; (**g**) single-band state with a passband at 9.6 GHz; (**h**) multiband state with two passbands at 4.8 GHz and 9.6 GHz.

The Lyot loop filter can be tuned to have multiple combs with different comb spacings simultaneously by setting Δ*θ* to a value between 0 and *π/*4, such that a higher order filter is resulted. As shown in Fig. 3d, two optical combs with a different comb spacing are observed by using a one-stage Lyot loop filter. This situation can be regarded as two optical combs with different spacings appearing at the same time and spectrally interleaved with one another, which makes it possible to generate a MWP filter with two passbands at the same time. Based on different comb spacing combinations, a MWP multiband filter with switchable frequency bands can be achieved: with a one-stage Lyot loop filter, an optical comb with 2 selectable spacings is obtained; when a second stage is added into the Lyot loop filter, up to 12 selectable spacings are achieved^41^, corresponding to a MWP filter with 12 different possible passbands. Table 1 shows the 12 combinations of the two-stage Lyot loop filter, as well as the calculated relationship between the equivalent length of PMF (*L*_*e*_) and the passband frequency of the MWP filter (*Ω*_*0*_). *L*_*1*_ and *L*_*2*_ are the physical lengths of the PMF in the first and second stage, respectively. In the experiment, both *L*_*1*_ and *L*_*2*_ are set such that all the 12 equivalent lengths are different and the resultant passbands are evenly distributed within the frequency range of interest (0–10 GHz).

## Switchable Microwave Photonic Multiband Bandpass Filter

We first investigate the performance of our proposed MWP multiband filter with a one-stage Lyot loop filter. The spectrally sliced Gaussian broadband optical source with different comb spacings are shown in Fig. 3a–3d, measured by an optical spectrum analyzer with a resolution of 0.8 pm. A piece of 12-m PMF with a birefringence of 6.6 × 10^−4^ is used in the one-stage Lyot loop filter. Through polarization state adjustment, 30-nm wide Gaussian optical combs with four different comb spacing combinations are observed (0.66 nm, 0.33 nm and interleaved 0.66 nm & 0.33 nm), corresponding to a PMF equivalent length of 0m, 12 m, 24 m, and interleaved 12 m & 24 m, respectively. The extinction ratios of the optical combs in Fig. 3a–d are over 20 dB for all combinations. RF spectra of the resultant MWP filter generated from the corresponding optical comb are shown in Fig. 3e–h, measured by a RF network analyzer with an intermediate frequency bandwidth of 5 kHz. In Fig. 3e, no passband is observed and the MWP filter is working in the “all-block” state, corresponding to a polarization rotation angle Δ*θ* of π/2. While in Fig. 3f and g, the filter is in a “single-band” state that only consists of one passband at either 4.8 GHz or 9.6 GHz (Δθ = π/4 and 0, respectively). By properly adjusting the PC in the circulator-PC loop one can achieve two different comb spacings at the same time (Δ*θ* is between 0 and *π/*4). Two clean passbands are observed and the MWP filter is in “multiband” state, as shown in Fig. 3h. The four different configurations of the MWP filter are switchable by adjusting the PC to different polarization rotation angles. The 30-nm wide Gaussian profile optical comb provides enough taps for the MWP filter, resulting in good sidelobe suppression and a clean filter profile. All the passbands show consistent performance, with a sidelobe suppression of 46 dB and a 3-dB bandwidth of 200 MHz.

**Figure 4:**
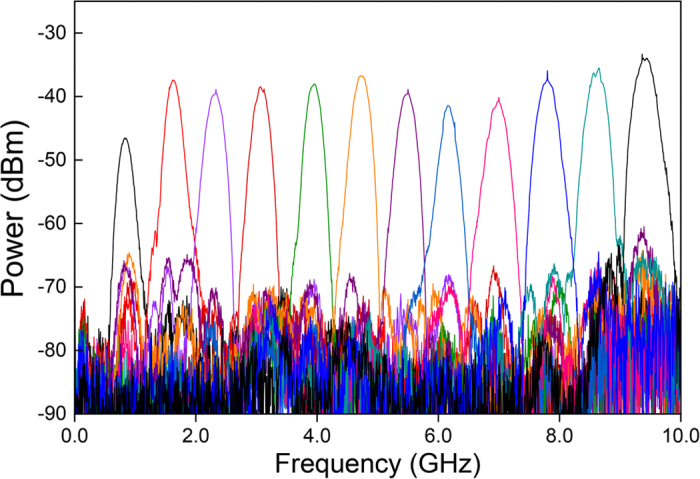
Measured frequency spectra of the MWP filter in single-band state. 12 single passbands are evenly distributed from 0.8 GHz to 9.6 GHz, with sidelobe suppression of 40 dB and 3-dB bandwidth of 200 MHz.

With a two-stage Lyot loop filter, 12 different passband center frequencies can be obtained from the proposed MWP filter. Two pieces of PMFs with lengths of 2 m (*L*_*1*_) and 10 m (*L*_*2*_) are used in each state of the Lyot loop filter. The lengths of the PMFs are chosen to make the equivalent length (*L*_*e*_) to have the same length difference between adjacent combinations, which results in an even frequency distribution of all the possible passbands, as shown in Table 1. First, the MWP filter is set to operate in the single-band state, i.e. only one passband appears at one time. Figure 4 shows the measured RF frequency spectra of all the 12 different single passband outputs of the MWP filter, i.e. from 12 different measurements. The single passband of the MWP filter is tuned to 12 different frequency positions through polarization state adjustment in the two-stage Lyot loop filter. The 12 passbands are evenly distributed from 0 to 10 GHz with a same frequency spacing of 0.8 GHz, and the experimental result agrees well with the calculation in Table 1. The passband has a 3-dB bandwidth of 200 MHz and the sidelobe suppression is up to 40 dB, with a sharp and clean filter profile. It is worth noticing that the sidelobe suppression of the first passband at 0.8 GHz is about 30 dB, which is smaller than other passbands. This is due to the fact that the corresponding comb spacing of the first passband is relatively large, such that there is not enough comb lines (taps) for the MWP filter, which results in a relatively lower sidelobe suppression.

**Figure 5:**
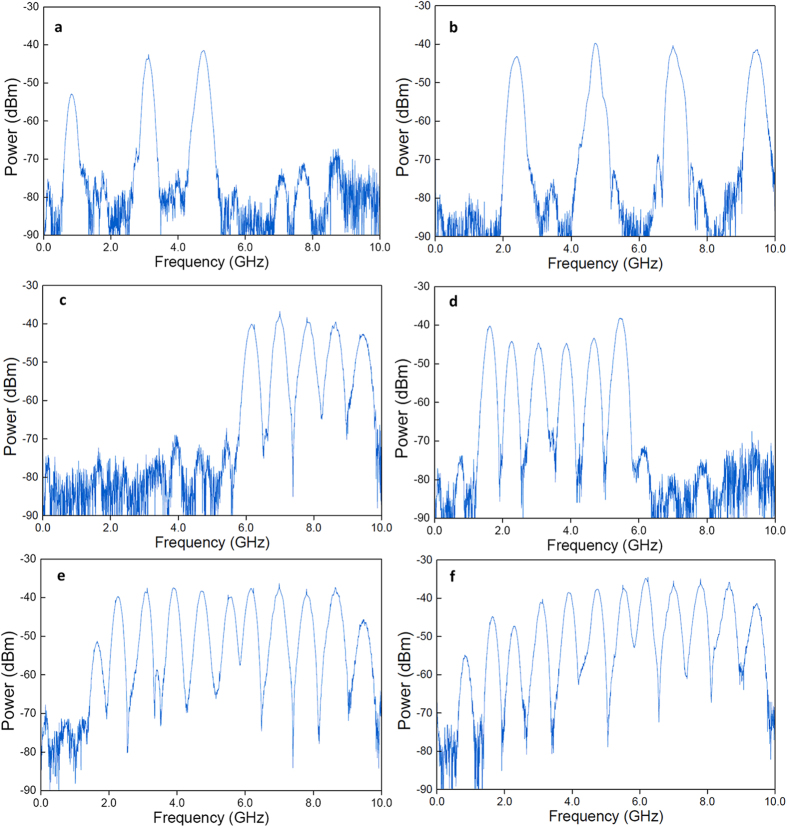
Measured frequency spectra of the MWP filter in multiband state with different combinations of passbands. (**a**) Three passbands at 0.8 GHz, 3.2 GHz and 4.8 GHz. (**b**) Four passbands at 2.4 GHz, 4.8 GHz, 7.2 GHz and 9.6 GHz. (**c**) Five passbands at 6.4 GHz, 7.2 GHz, 8.0 GHz, 8.8 GHz and 9.6 GHz. (**d**) Six passbands at 1.6 GHz, 2.4 GHz, 3.2 GHz, 4.0 GHz, 4.8 GHz and 5.6 GHz. (**e**) Eleven passbands from 1.6 GHz to 9.6 GHz. (**f**) Twelve passbands from 0.8 GHz to 9.6 GHz.

Frequency spectra of the proposed MWP multiband filter working in the multiband state are shown in Fig. 5. By setting the two-stage Lyot loop filter to have more than one comb spacing combination interleaving spectrally, a MWP filter with multiple passbands is achieved. With the adjustment of Δ*θ*, various combinations of the number of passbands and passband frequencies are achieved. In Fig. 5a, three passbands at 0.8 GHz, 3.2 GHz and 4.8 GHz are obtained simultaneously from the MWP multiband filter, while four passbands at 2.4 GHz, 4.8 GHz, 7.2 GHz and 9.6 GHz are observed in Fig. 5b. In Fig. 5c–e, five, six and eleven passbands from 6.4–9.6 GHz, 1.6–5.6 GHz and 1.6–9.6 GHz are obtained, respectively. All the 12 passbands of the MWP multiband filter calculated in Table. 1 are observed at the same time as shown in Fig. 5f, with an equal frequency spacing of 0.8 GHz between each passband. Sidelobe suppression is over 30 dB for all the passbands except the first passband at 0.8 GHz, as explained previously. Noticing that when adjacent passbands are chosen, the transition bandwidth between pass and stop bands are smaller. This can be improved by using a longer DCF or DCF with a higher dispersion, such that a narrower passband and wider transition bandwidth are resulted. Compared with current state-of-the-art multiband RF filters, the proposed MWP multiband filter has uniform passband response, flexible reconfiguration capability and a much larger number of simultaneous passbands. Since the passband selection is based on polarization state adjustment in the Lyot loop filter, systematic and high-speed tuning of the passband numbers and passband frequencies can be potentially achieved at kilohertz to gigahertz speed with the use of an electrically tunable polarization controller or optical nonlinear polarization rotation in nonlinear devices^42^,^43^. Miniaturization of the MWP multiband filter could be achieved with photonic crystal waveguide technology^17^ and integrated photonic technology^44^,^45^.

## Conclusion

In summary, a MWP multiband filter with reconfigurable passband numbers and center frequencies is experimentally demonstrated. Up to 12 different passbands can be selected simultaneously and are evenly distributed from 0 to 10 GHz. The adjustment of passband numbers and center frequencies are achieved through polarization state adjustment in the two-stage Lyot loop filter. Sidelobe suppression of all the passbands is over 30 dB and each passband has a 3-dB bandwidth of 200 MHz with a sharp and clean bandpass profile, providing good filter selectivity. Moreover, the proposed MWP multiband filter can work either as an all-block, single-band, or multiband RF filter with adjustable multi-passbands combinations. This design significantly increases the number of passbands that can be achieved with a MWP multiband filter as well as providing exceptional operation flexibility. To the best of our knowledge, this is the first demonstration to achieve a MWP bandpass filter with more than six tunable passbands simultaneously and with such exceptional operation flexibility, which is extremely useful for multiband signal multiplexing applications. With the proposed reconfigurable MWP multiband filter, the capabilities of multiband wireless systems and satellite systems could be fully exploited. Thus, the systems could be operated at various frequency bands in diverse environments, and the operating frequencies could be switched dynamically according to the required functionality, spectral availability, and channel performance.

## Additional Information

**How to cite this article**: Ge, J. and Fok, M. P. Passband switchable microwave photonic multiband filter. *Sci. Rep.* **5**, 15882; doi: 10.1038/srep15882 (2015).

## Figures and Tables

**Table 1:** 12 combinations of the two-stage Lyot loop filter based MWP multiband filter (L_1_ = 2 m, L_2_  = 10 m).

Number	Length Combination	Equivalent PMF length (L_e_, m)	MWP Filter passband (Ω_0_, GHz)
1	L_1_	2	0.8
2	2L_1_	4	1.6
3	|2L_1_–L_2_|	6	2.4
4	|L_1_–L_2_|	8	3.2
5	L_2_	10	4.0
6	L_1_+L_2_	12	4.8
7	2L_1_+L_2_	14	5.6
8	|2L_1_–2L_2_|	16	6.4
9	|L_1_–2L_2_|	18	7.2
10	2L_2_	20	8.0
11	L_1_+2L_2_	22	8.8
12	2L_1_+2L_2_	24	9.6
